# Optimum Power Loads for Elite Boxers: Case Study with the Brazilian National Olympic Team

**DOI:** 10.3390/sports6030095

**Published:** 2018-09-13

**Authors:** Irineu Loturco, Chris Bishop, Rodrigo Ramirez-Campillo, Felipe Romano, Mateus Alves, Lucas A. Pereira, Michael McGuigan

**Affiliations:** 1 NAR—Nucleus of High Performance in Sport, São Paulo 04753060, Brazil; lucasa_pereira@outlook.com; 2 Faculty of Science and Technology, London Sports Institute, Middlesex University, London NW4 4BT, UK; C.Bishop@mdx.ac.uk; 3 Department of Physical Activity Sciences, Research Nucleus in Health, Physical Activity and Sport, Universidad de Los Lagos Osorno 5290000, Chile; r.ramirez@ulagos.cl; 4 Brazilian Boxing Confederation, São Paulo 11740000, Brazil; felipecbboxe@gmail.com (F.R.); mateus.alvescbb@gmail.com (M.A.); 5 Sports Performance Research Institute New Zealand (SPRINZ), Auckland University of Technology, Auckland 92006, New Zealand; michael.mcguigan@aut.ac.nz; 6 School of Medical and Health Sciences, Edith Cowan University, Perth, WA 6027, Australia

**Keywords:** elite athletes, combat sports, physical performance, power training, muscle power

## Abstract

The purpose of this case study was to examine the effects of a resistance-training program based on the optimum power loads (OPL) method on neuromuscular performance of Olympic boxing athletes during preparation for the Rio-2016 Olympic Games. Twelve elite amateur boxers from the Brazilian National Olympic Team participated in this study. Athletes were assessed at four time-points, over two consecutive competitive seasons. In the first season (considered as “control period”), the athletes executed a non-controlled strength-power training program for 10 weeks. In the second season (a seven-week experimental period), the elite boxers performed 14 power-oriented training sessions, comprising bench press (BP) and jump squat (JS) exercises at the OPL. Maximum bar-power output in BP and JS exercises was measured pre and post both training phases. Magnitude-based inferences were used to compare changes in pre and post training tests. Bar-power outputs increased meaningfully in both BP (+8%) and JS (+7%) exercises after the OPL training program. In contrast, after the control period, no worthwhile improvements were observed in the variables tested. Based on the findings of this study, highly trained boxers might benefit from the use of a training scheme based on OPL.

## 1. Introduction

Improving power output seems to be an essential and critical requirement in many sports. In this regard, several studies have shown that substantial increases in muscle power may also result in significant enhancements in performance, both in individual and team sports [1,2,3,4,5]. Furthermore, total power production is strongly related to the success obtained in specific sport tasks, such as jumps [1,4], maximal short-sprints [2,3,6], throwing velocity [7] and others which involve complex and increased physical contact (e.g., rugby tackling and scrummaging, and combat strikes) [8,9,10]. Therefore, coaches and researchers are constantly seeking better and more effective methods for developing muscle power in athletes.

More recently, the optimum power load (i.e., load capable of maximizing power output, (OPL)) has been shown to be a practical and effective way to acutely increase speed, strength, and power production in athletes from different sports [1,2,11]. For example, Dello Iacono and Seitz [11] compared the acute effects (i.e., post activation potentiation) of heavy (i.e., 85% 1RM) versus OPL hip thrust exercises on the sprint performance of young soccer players, reporting meaningfully greater increases in favor of the OPL protocol [2]. Similarly, in another study performed with professional soccer players, Loturco et al. [2] showed that the OPL method yielded significant increases in strength and power capacities, and superior improvements in overall speed performance (from 5 to 20 m) when compared with a traditional resistance training program (using loads varying from 30% to 90% 1RM). Despite these promising findings, there is a lack of research evaluating the effectiveness of OPL intervention strategies in the physical performance of athletes from individual sports such as combat athletes.

Specifically, in combat sports, a strong relationship has been observed between the amount of power generated at the OPL in certain exercises such as bench press (BP) and jump squat (JS) and striking efficiency [9,10]. In this context, it was revealed that top-level karate athletes that were able to produce higher levels of muscle power in bench throw and JS exercises were also able to punch faster from fixed distances [9]. The same holds true for National boxing athletes, who produced higher impact forces in jabs and crosses as long as they could generate more power in BP, bench throw, and JS exercises (r = 0.70 to 0.85, for all punching conditions) [10]. Taking into consideration that punching impact force is one of the main performance indicators in boxing [12,13,14], it would be interesting to investigate if a training scheme based on the OPL would increase power output in Olympic level boxing athletes.

Therefore, the purpose of this case report was to examine the effect of a resistance-training program based on OPL on neuromuscular qualities of Olympic boxing athletes during their final stages of preparation for the Rio-2016 Olympic Games. In addition, we compared the changes in performance obtained after this supervised training period with those obtained after a previous and unsupervised strength-power training period. It was hypothesized that there would be meaningful improvements in power performance after a controlled seven-week training program.

## 2. Materials and Methods

### 2.1. Study Design

Athletes were assessed at four time-points, across two consecutive competitive seasons. In the first season, athletes were assessed twice during a preparatory period for the 2015 Pan-American Games (Toronto 2015), comprising a 10-week training period. This was the “control” period, as the athletes’ strength-power training program was not controlled. In the second season, physical tests were carried out, pre and post 7 weeks of training, with the same athletes, during preparation for the 2016 Olympic Games (Rio 2016). During this experimental period, athletes performed 14 power-oriented training sessions comprising BP and JS exercises with their OPL. The schematic presentation of the training schedule during the power-training period is presented in Table 1. During the control period, the strength-power training program served as a complementary activity and was not controlled. In this period, coaches reported that they commonly used moderate-to-heavy loads in several exercises also comprising BP and JS exercises. Therefore, this comparison allows better understanding of the effectiveness of a supervised power-oriented training regime.

The maximum bar-power outputs (i.e., mean power (MP), mean propulsive power (MPP), and peak power (PP)) in BP and JS exercises were assessed pre and post training in both seasons. Subjects were instructed to arrive at the sports laboratory in a fasted state for at least 2 h. Due to their regular assessments in our facilities, all subjects had been previously familiarized with the testing procedures. A standardized warm-up comprising light to moderate self-selected running for 5 min was performed before the tests. Sub-maximal attempts (using an unloaded Smith-machine bar weighing 20 kg) at each test were also executed prior to the maximal tests. All physical tests were performed between 8:00 a.m. and 12:00 p.m. on all occasions.

### 2.2. Participants

Twelve elite amateur boxers from the Brazilian National Olympic Team (7 men, age: 27.9 ± 4.2 years; weight: 74.6 ± 18.3 kg; height: 175.3 ± 9.5 cm; 5 women, age: 28.4 ± 3.6 years; weight: 63.6 ± 10.6 kg; height 165.2 ± 3.3 cm) participated in this study. The participants included one Olympic champion, one Olympic bronze medalist, and three Pan-American Games medalists, and all athletes were South-American Games medalists. Prior to participating in this study, athletes were briefed on the experimental design and signed an informed consent form. This study was performed in accordance with the ethical standards of the Helsinki Declaration and was approved by the local Ethics Committee.

### 2.3. Bar-Mean, Mean Propulsive, and Peak Power in Jump Squat and Bench Press Exercises

**Figure 1:**
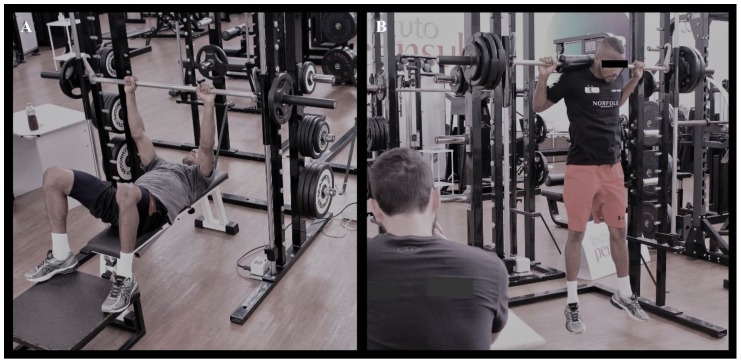
An Olympic champion performing bench press (**A**) and jump squat (**B**) exercises at the optimum power zone.

Maximum bar-power outputs were assessed in JS and BP (Figure 1), with all being performed on a Smith-machine (Hammer Strength Equipment, Rosemont, IL, USA). Participants were instructed to execute three repetitions at maximal velocity for each load, starting at 40% (27.5 ± 6.4 kg) of their body mass (BM) in the JS and 30% (22.5 ± 3.5 kg) of their BM in the BP. In the JS, participants executed knee flexion until the thigh was parallel to the ground and, after the command to start, jumped as fast as possible without their shoulders losing contact with the bar. During the BP, athletes were instructed to lower the bar in a controlled manner until the bar lightly touched the chest and, after the command, moved the bar as fast as possible. A load of 10% of BM for JS and 5% of BM for BP was progressively added for each set until a clear decrement (5%) in MP, MPP, and PP were observed [15]. A 5-min rest period occurred between sets. To determine the power outputs, a linear position transducer (T-Force, Dynamic Measurement System; Ergotech Consulting S.L., Murcia, Spain) was attached to the Smith-machine bar and values were automatically derived by the custom-designed software as follows: MP—value calculated during the entire concentric phase of each repetition; MPP—value calculated during the propulsive phase, defined as that portion of the concentric action during which the measured acceleration is greater than acceleration due to gravity; PP—the highest bar-power value registered at a particular instant (1-ms) during the concentric phase [16,17]. The bar position data were sampled at 1000 Hz. The maximum MP, MPP, and PP values obtained in each exercise were used for analysis. Values were normalized by dividing the absolute power by the athletes’ BM (i.e., relative power = W·kg^−1^). Both BP and JS bar-power measures presented good levels of absolute and relative reliability (CV < 5% and ICC > 0.90, for all assessments) [18].

### 2.4. Statistical Analysis

Data are presented as mean ± 90% confidence limits (CL). The magnitude-based inferences method was used to analyze the differences in the bar-power outputs in the BP and JS exercises in both control and power-oriented training programs, pre- and post-training [19]. This method was chosen in place of traditional null-hypothesis testing methods based on an arbitrary *p* value as it allows emphasis to be placed on the effect magnitudes and estimated precision, focusing on non-effect interpretation rather than on absolute effect [19]. In addition, the traditional method does not deal with the real world significance of an outcome [19,20], whilst the magnitude-based method defines the practical effect, allowing the researcher to quantify the probability of a worthwhile effect with inferential descriptors to aid interpretation [19,20]. Whereas traditional inferential statistics can be misleading, depending on the magnitude of the statistic, error of measurement, and sample size [19,21], magnitude-based inferences recognize sample variability, and provide scientists and coaches with an indication of the practical meaningfulness of the outcomes. Points and counterpoints in relation to this statistical method have been discussed elsewhere [21,22,23,24]. The quantitative chances of both training periods having higher, similar, or lower values were assessed qualitatively as follows: <1%, almost certainly not; 1% to 5%, very unlikely; 5% to 25%, unlikely; 25% to 75%, possible; 75% to 95%, likely; 95% to 99%, very likely; >99%, almost certain [17]. If the chances of having better and poorer results were both >5%, the true difference was assessed as unclear. Additionally, to determine the magnitude of the differences between the two periods, pre- and post-training, and the delta changes, the standardized mean differences (Cohen’s *d* effect sizes (ES)) were calculated [18]. The ES magnitudes were interpreted using the following thresholds: <0.2, 0.2–0.6, 0.6–1.2, 1.2–2.0, 2.0–4.0, and >4.0 for trivial, small, moderate, large, very large, and near perfect, respectively [18]. The original spreadsheet designed by Hopkins [20] was used for data analysis.

## 3. Results

**Figure 2:**
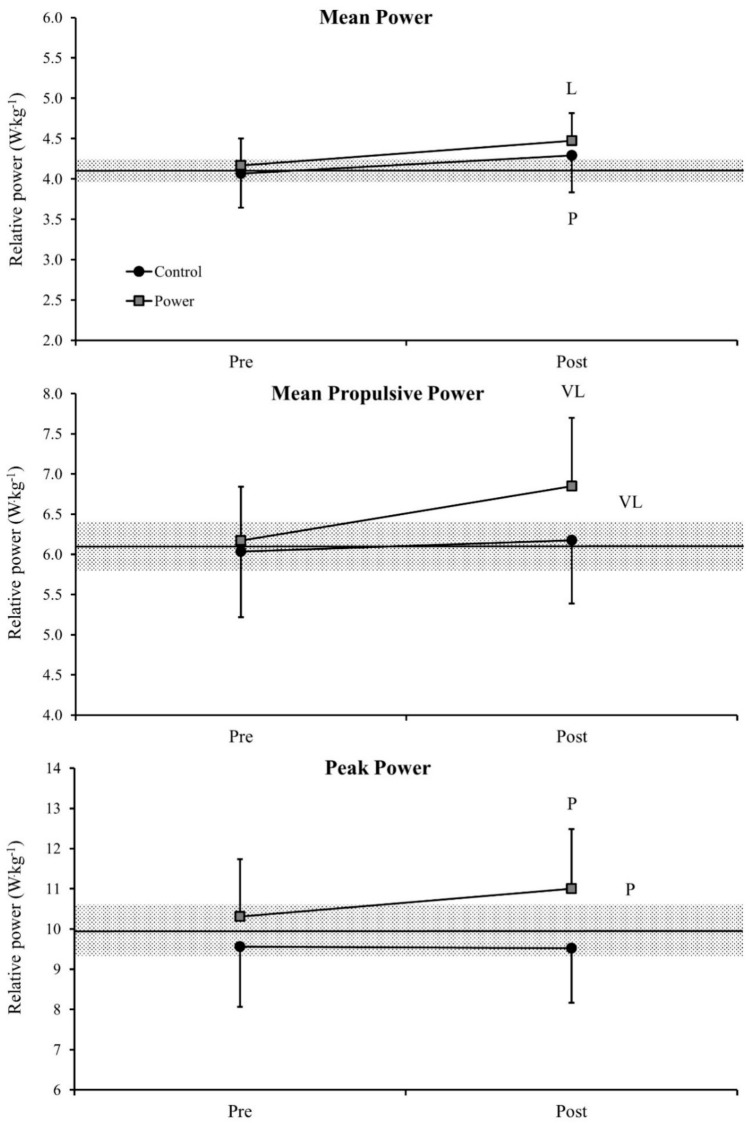
Comparison of relative bar-power outputs in the bench press exercise pre and post training and comparisons of delta changes between the two different competitive seasons. Values are presented as mean ±90% confidence limits. Middle horizontal lines represent mean pre-test values from both seasons. The gray area represents the smallest worthwhile change (calculated using 0.2× pre-values standard deviation). P = possible difference, L = likely difference, VL = very likely difference. Letters indicating differences comparing pre- and post-measures are presented above and below the bar errors, while the differences comparing delta changes are presented on the right side. The other comparisons did not show meaningful differences.

No meaningful differences were observed in the weight of the athletes comparing both periods of training. Figure 2 shows the bar-power changes in the BP exercise pre and post training and the comparisons of delta changes between the two different competitive seasons. A likely and a possible change in MP were observed for power (ES = 0.42) and control (ES = 0.25) periods, respectively. For the MPP a very likely improvement was observed in the power period (ES = 0.46) with a very likely difference being observed between the delta changes comparing both phases (ES = 0.33). A possible difference was observed in the PP when comparing pre and post training assessments in the power period (ES = 0.22), which was also followed by a possible difference in the delta changes comparing both observed seasons (ES = 0.24). The differences between pre- and post-measures in the control period for MPP and PP, as well as the differences in the delta changes comparing both phases for the MP values were all unclear.

**Figure 3:**
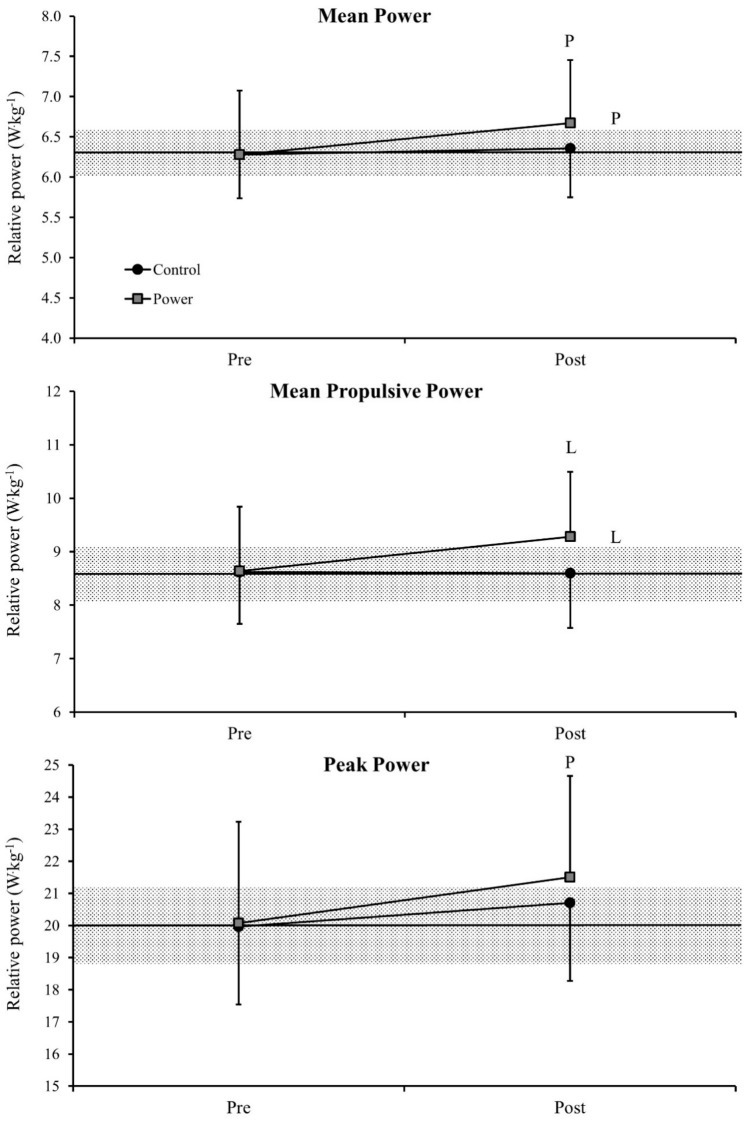
Comparison of the relative bar-power outputs in the jump squat exercise pre and post training and comparisons of delta changes between the two different competitive seasons. Values are presented as mean ±90% confidence limits. Middle horizontal lines represent mean pre-test values from both seasons. The grey area represents the smallest worthwhile change (calculated using 0.2× pre-values standard deviation). P = possible difference, L = likely difference. Letters indicating differences comparing pre- and post-measures are presented above and below the bar errors, while the differences comparing delta changes are presented on the right side. The other comparisons did not show meaningful differences.

Figure 3 demonstrates bar-power changes in the JS exercise pre and post training and the comparisons of delta changes between the two different competitive seasons. A possible difference was observed in the MP when comparing pre and post training assessments in the power period (ES = 0.22), which was also followed by a possible difference in the delta changes comparing both observed seasons (ES = 0.24). For the MPP, a likely difference was observed between pre and post training assessments in the power period (ES = 0.23), with a likely difference also being observed between the delta changes comparing both periods (ES = 0.30). Finally, a possible difference was observed in the PP when comparing pre and post training assessments in the power period (ES = 0.20). The differences between pre- and post-measures in the control period for all power outputs, as well as the differences in the delta changes comparing both periods for the PP were all unclear.

## 4. Discussion

This case study examined the effects of a seven-week OPL training scheme on the physical performance of Olympic boxing athletes. After comparing the pre- and post-testing measures, we verified that power output increased meaningfully in both BP and JS exercises after the power-oriented training period based on the OPL. In addition, when analyzing the changes during both training phases, meaningful differences (in both exercises) were observed in favor of the OPL group. Conversely, after the control period, we did not detect any worthwhile increases in the assessed variables. These results are similar to those previously described in team sport athletes [2,25,26]. To our knowledge, this is the first study to report these benefits in Olympic athletes from the individual sport of boxing. Based on these findings, it can be concluded that highly trained boxers might benefit from the use of a short-term training program based on the loads that maximize power production.

From an applied perspective, meaningful increases in power output in both BP (~8%) and JS (~7%) exercises may have a positive influence on boxing performance. In fact, the power produced by combat athletes in these exercises has been shown to be closely related to punching impact and acceleration [9,10]. Furthermore, it was demonstrated that, when compared to National Team peers, a double world karate champion generated 45% and 7% more power in JS and BP, respectively [27]. In boxing, achieving a knockout is a continuous goal throughout a fight; as such, top-level boxers must optimize their strength-power capabilities to improve punching impact force and, therefore, knockout power [10,11,14,28]. Based on these observations, it is possible to speculate that at the same level of specialization (National Team), the more powerful athletes are also more likely to achieve the best results in important competitions. For example, the male Olympic champion in this sample (Rio-2016) could produce (on average) 49% and 15% more power than his male National Team peers, respectively, in JS and BP exercises.

The opportunity to compare the seven-week experimental period with a control period (10 weeks without a controlled training program) has particular relevance in this case study. Although it was not possible to accurately define the volume and intensity of the strength-power training during the control phase, the coaches reported that the boxers frequently performed BP and JS in their training sessions, between two and three times per week, using moderate to moderate-heavy loads. According to the coaches, the remaining training content (e.g., technical and conditioning training activities) was very similar in both periods. Therefore, we can infer that the increases in power output were a result of the implementation of the OPL training scheme. These data are consistent with previous research indicating that the OPL can be effectively used to improve the competitive performance of elite athletes from different sports [1,2,5,11,29].

We recognize the limitations of this case study, such as the lack of control or other treatment groups, and small sample-size. Nevertheless, it is important to emphasize that the study was composed by a highly specialized sample of elite boxing athletes (i.e., Olympic medalists). In theory, this should also mean that their window of opportunity [30] for training adaptation should be less; thus, gaining that extra 7%–8% improvement in terms of power production could be of great importance for elite boxing performance. These findings provide better understanding of these unique subjects, and generate more effective communication between coaches and researchers, which is an important requirement in sport science [31,32,33]. 

## 5. Conclusions

A short-term OPL training program (seven-week) composed of BP and JS exercises performed two or three times per week was able to produce meaningful increases in power output in Olympic National Team boxers. Due to the strong relationships observed between BP and JS power measures and punching impact, boxing coaches and practitioners are strongly encouraged to use the OPL training approach with their professional athletes.

## Figures and Tables

**Table 1:** Schematic presentation of the training schedule * during the power-oriented training regime of elite amateur National Team boxers.

	Monday	Tuesday	Wednesday	Thursday	Friday
**Week 1**	-	-	Physical Tests	Conditioning	Power Training 4 × 6 (BP–JS)
**Week 2**	Conditioning	Power Training 4 × 6 (BP–JS)	Conditioning	Power Training 4 × 6 (BP–JS)	Rest
**Week 3**	Power Training 6 × 6 (BP–JS)	Conditioning	Power Training 6 × 6 (BP–JS)	Conditioning	Power Training 6 × 6 (BP–JS)
**Week 4**	Power Training 6 × 6 (BP–JS)	Conditioning	Power Training 6 × 6 (BP–JS)	Conditioning	Physical Tests ^#^
**Week 5**	Power Training 4 × 6 (BP–JS)	Conditioning	Power Training 4 × 6 (BP–JS)	Conditioning	Power Training 4 × 6 (BP–JS)
**Week 6**	Conditioning	Power Training 4 × 4 (BP-JS)	Conditioning	Power Training 4 × 4 (BP–JS)	Rest
**Week 7**	Power Training 4 × 4 (BP–JS)	Rest	Physical Tests	-	-

Note: Conditioning training involved ~30/40 min of circuit training, running and/or jump rope. * Physical training sessions were all performed in the morning, while technical training sessions involving specific punching technique and sparring, were performed in the afternoon, lasting between 60 and 120 min. ^#^ Physical tests were performed in the 4th week to adjust the training loads. BP: bench press; JS: jump squat.
